# Epidemiology of tuberculosis in the Pacific island countries and areas, 2000–2020

**DOI:** 10.5365/wpsar.2023.14.1.996

**Published:** 2023-02-22

**Authors:** Manami Yanagawa, Fukushi Morishita, Kyung Hyun Oh, Kalpeshsinh Rahevar, Tauhidul Abm Islam, Subhash Yadav

**Affiliations:** a World Health Organization Regional Office for the Western Pacific, Manila, Philippines.; b World Health Organization, Geneva, Switzerland.; c Division of Pacific Technical Support, World Health Organization, Suva, Fiji.

## Abstract

**Objective:**

Tuberculosis (TB) is one of the most important infectious diseases with an estimated 9.9 million people falling ill globally in 2020. We describe the epidemiology of TB in the Pacific island countries and areas (PICs) to inform potential priority actions to implement the *Western Pacific Regional Framework to End TB 2021–2030*.

**Methods:**

A descriptive analysis was conducted using annual TB surveillance data submitted by national TB programmes to the World Health Organization (WHO) and TB burden estimates (incidence rates and number of deaths) generated by WHO for the PICs, for the period 2000–2020. We also analysed TB case numbers, multidrug-resistant/rifampicin-resistant TB (MDR/RR-TB), recent risk factor indicators and treatment outcomes.

**Results:**

The estimated TB incidence rate in the PICs increased between 2000 and 2020 from 62 to 69 per 100 000 population, with an 8% reduction observed since 2015. TB cases increased by 29% during 2000–2020, with 1746 cases in 2020 and a high proportion in children (19%). Bacteriological diagnosis was used for 58% of total TB cases, although some countries reported clinical diagnoses in over 60% of cases. From 2015 to 2019, 52 MDR/RR-TB cases were reported and there were 94 TB/HIV coinfected cases in 2015–2020. Treatment success was 74% in 2019 due to 18% of cases being unevaluated. In 2020, the estimated proportion of TB cases attributable to smoking, malnutrition, alcohol abuse and diabetes was 17%, 16%, 11% and 9%, respectively.

**Discussion:**

There was an increasing trend in TB cases, estimated incidence and deaths between 2000 and 2020. Laboratory services were scaled up in some PICs and case-finding activities greatly contributed to the detection of cases. To end the incidence of TB, continued efforts on case finding, contact investigation and scaling up TB preventive treatment should be prioritized. At the same time, collaboration with other sectors for risk factor management and decentralized management need to be considered.

Tuberculosis (TB) remains a disease with a major global impact. In 2020, an estimated 9.9 million people developed TB; there were an estimated 1.3 million deaths among human immunodeficiency virus (HIV)-negative TB cases and an estimated 214 000 deaths among HIV-positive TB cases reported worldwide. (*1*) The World Health Organization (WHO) Western Pacific Region, which consists of 37 countries and areas, accounted for 18% of these estimated cases. From 2015 to 2020, the estimated global TB incidence and number of deaths declined by 6% and 13%, respectively, with annual reductions of 1.2% and 2.6%, respectively.

The population of the 20 Pacific island countries and areas (PICs) of the Western Pacific Region included in this paper is approximately 3.4 million. The PICs are made up of around 1300 islands with limited transport services between them. (*2*, *3*) Economic status varies by country and area, with gross national income data available for six of the 16 PICs which are classified as high-income countries, while the remainder are classified as middle-income countries. (*4*) Twelve PICs are supported by the Global Fund to fight AIDS, Tuberculosis and Malaria, three of which are classified as high-income countries. (*5*)

The estimated TB incidence in the PICs is lower than other countries in the Western Pacific. (*6*) However, the epidemiology of TB is diverse, ranging from countries with high TB burdens to those in the pre-elimination stage (defined as < 10 TB cases per million). (*7*) Among the PICs, Kiribati, the Marshall Islands and the Federated States of Micronesia had a high estimated TB incidence per capita in 2020. (*8*) Some countries reported a low treatment success rate despite the relatively young age group affected. (*6*)

In 2015, the *Regional Framework for Action on Implementation of the End TB Strategy in the Western Pacific 2016–2020* was endorsed by the WHO Regional Committee for the Western Pacific, (*9*) following the release of the End TB Strategy. (*10*) Since then, recommended interventions from the Framework have been implemented in countries and areas to achieve the 2020 milestones and targets. The new *Western Pacific Regional Framework to End TB 2021–2030* was endorsed by the Regional Committee in October 2021. (*8*) The Framework is intended to support Member States in making further progress towards ending TB. In this paper, we describe the epidemiology of TB in the PICs by analysing existing TB surveillance and burden estimate data available in the WHO Global TB Programme for the period 2000–2020, focusing particularly on 2015 and 2020. The results may inform potential priority actions required to implement the *Western Pacific Regional Framework to End TB 2021–2030* in the PICs.

## Methods

This descriptive analysis used annual TB surveillance data submitted by national TB programmes to WHO and TB burden estimates (incidence and mortality) generated by WHO for the PICs for the period 2000–2020. This timeframe was selected as burden estimates were available for that period. A baseline of 2015 was used to monitor progress against the milestones and targets set by the End TB Strategy and the Regional Framework for 2016–2020.

Routine TB surveillance data were submitted annually by 20 national TB programmes in the PICs – American Samoa, the Cook Islands, Fiji, French Polynesia, Guam, Kiribati, the Marshall Islands, the Federated States of Micronesia, Nauru, New Caledonia, Niue, the Northern Mariana Islands, Palau, Samoa, the Solomon Islands, Tokelau, Tonga, Tuvalu, Vanuatu and Wallis and Futuna – referred to as a subregion in this paper. The Pitcairn Islands are excluded from annual TB data collection and are not included in the analysis. The verified data are published on the WHO web site in the annual *Global Tuberculosis Report 2022*, together with estimates of the TB disease burden, which are measured by incidence rates and deaths. (*11*) Methods used to estimate the TB disease burden are described in the technical annexes of the Report. (*12*)

The descriptive analysis included estimated incidence and deaths, case numbers (totals, by type of TB and diagnosis category), numbers of multidrug-resistant/rifampicin-resistant TB (MDR/RR-TB) cases detected and enrolled in multidrug-resistant tuberculosis (MDR-TB) treatment and key indicators of collaborative TB/HIV activities. The proportion of bacteriologically confirmed, clinically diagnosed pulmonary TB (PTB) and extra-pulmonary TB (EPTB) cases, as well as age and sex distributions and treatment outcomes, were compared across countries and areas. The proportion of TB cases attributable to alcohol abuse, diabetes mellitus, smoking and undernutrition were also analysed. The estimated numbers of cases attributable to these risk factors generated by WHO data were used to make an overall comparison of the cases in the PICs and the Western Pacific Region. The number of PICs with these estimations is limited and, therefore, the risk factors vary.

The definitions of cases and treatment outcomes were in accordance with the WHO reporting framework for TB. (*13*) The diagnosis category was changed in 2013 when new case definitions of bacteriologically confirmed TB and clinically diagnosed TB were introduced to replace smear-positive and smear-negative TB, respectively, to align with the increased availability of Xpert testing. Most of the analyses, such as age and sex distribution, HIV testing data and treatment outcomes, are of incident cases which were redefined as “new and relapse (or previous history unknown)” cases, regardless of bacteriological confirmation.

Estimated incidence and deaths, 95% confidence intervals (CI) and total case numbers for the subregion were calculated by aggregating existing burden estimates and data from each country and area. We used population estimates from the United Nations Population Division to calculate rates per capita where required. Data analyses and visualization were conducted with the statistical software package R 4.1.2 (Comprehensive R Archive Network: https://cran.r-project.org/). Only those countries and areas that had data for each variable, with more than five cases between 2015 and 2020, were included in the analyses.

## Results

### Estimates of TB burden

**Fig. 1:**
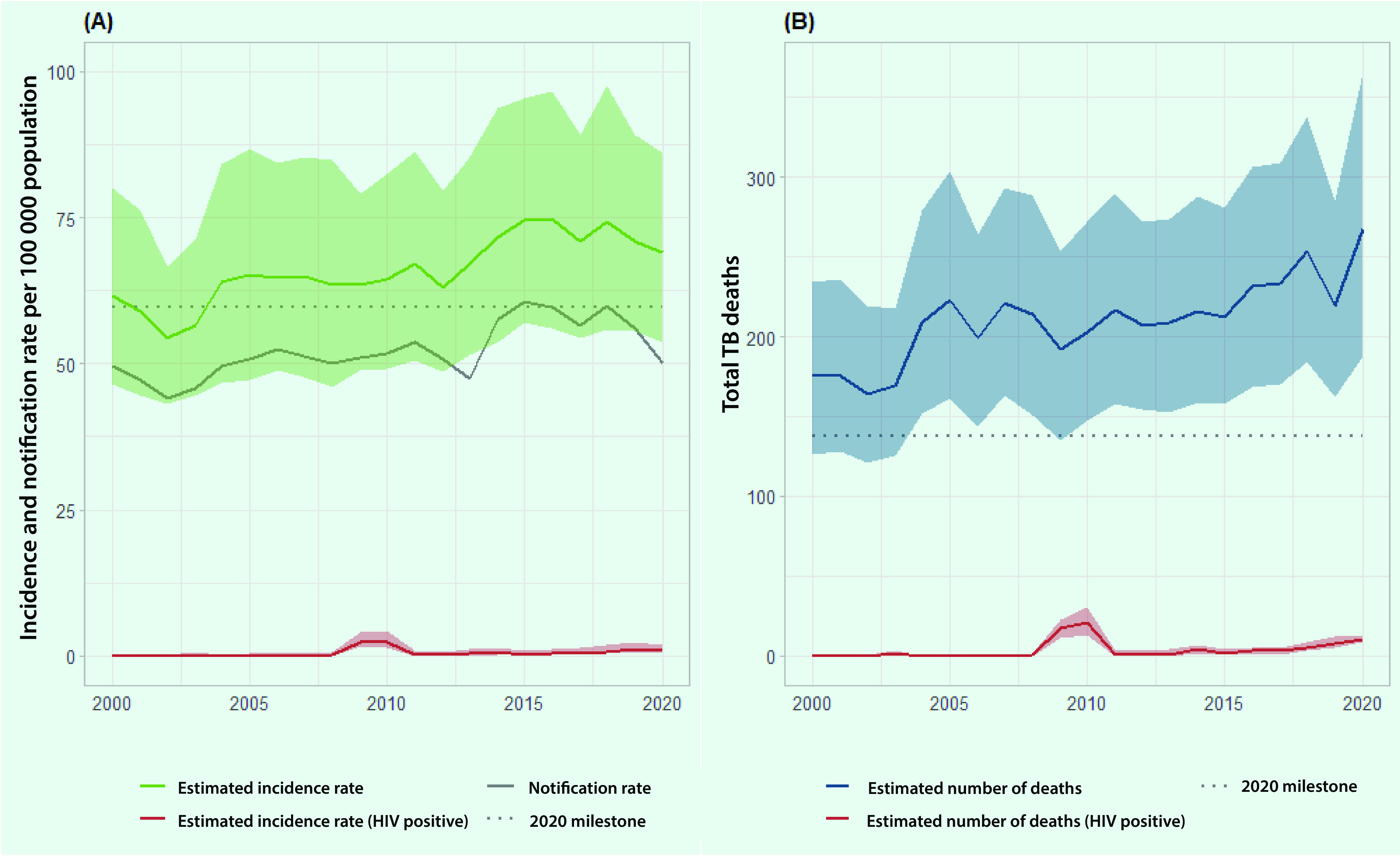
(A) Estimated TB incidence and notification rates of new and relapse TB cases, and (B) estimated number of TB deaths in Pacific island countries and areas (including among people living with HIV), 2000–2020

The estimated incidence rate of TB in the subregion increased from 62 (95% CI: 46–80) per 100 000 population in 2000 to 75 (95% CI: 57–96) per 100 000 population in 2015, before decreasing to 69 (95% CI: 54–86) per 100 000 population in 2020 (**Fig. 1A**). This equates to an estimated 1680 cases (95% CI: 1266–2185) in 2000, 2390 cases (95% CI: 1825–3061) in 2015 and 2356 cases (95% CI: 1827–2936) in 2020. The estimated number of TB deaths increased from 176 (95% CI: 126–234) in 2000 to 212 (95% CI: 158–281) in 2015 and further increased to 268 (95% CI: 188–366) in 2020 (**Fig. 1B**).

[**insert** Figure 1]

The estimated TB incidence rate and the number of deaths among people living with HIV (PLHIV) per 100 000 population in 2020 have remained low in the PICs at 0.9 (95% CI: 0.5–2.0) and 10 (95% CI: 9–13), respectively.

**Fig. 2:**
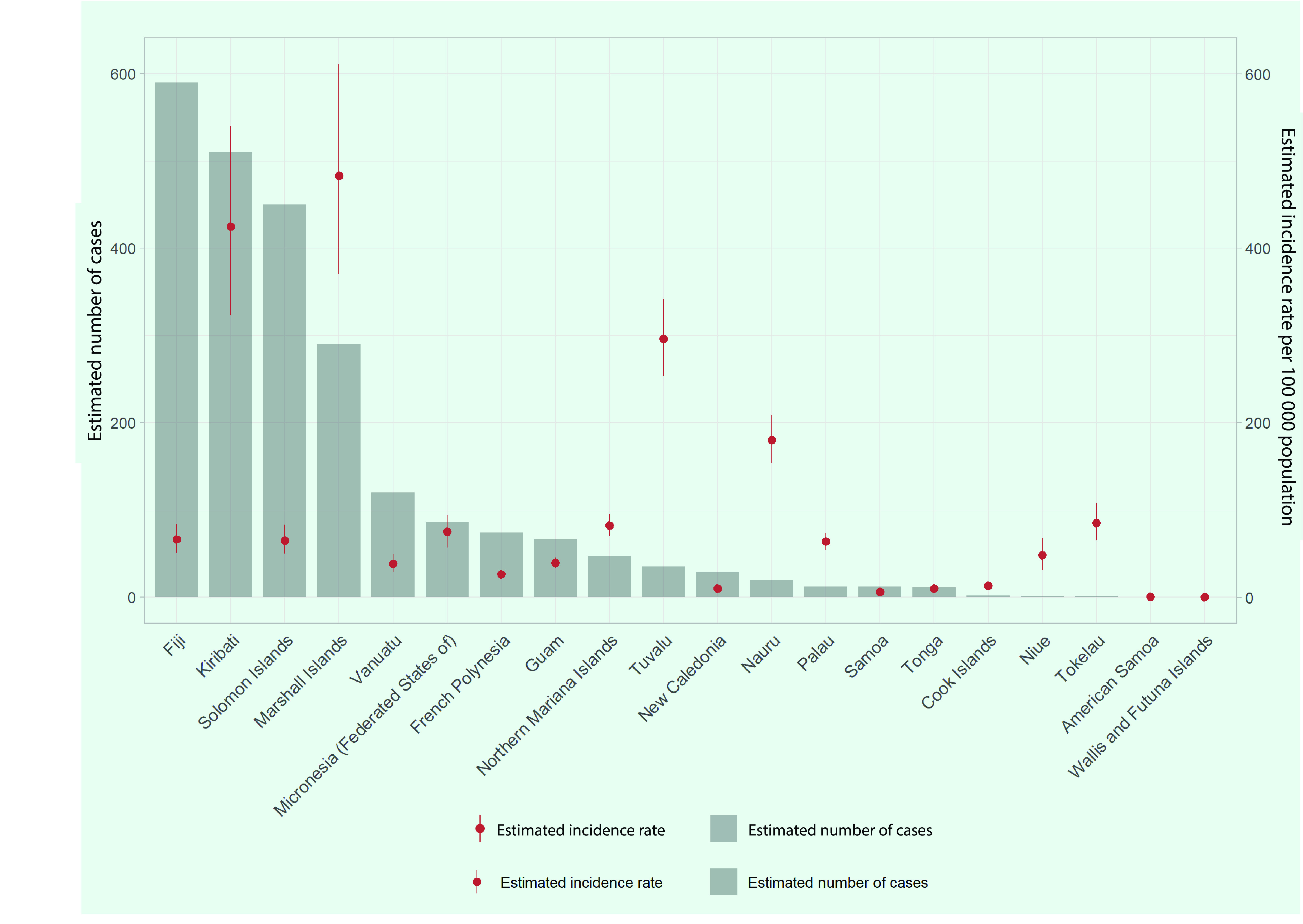
Estimated number of TB cases and TB incidence rate per 100 000 population in Pacific island countries and areas, 2020

By country and area, the Marshall Islands and Kiribati had the highest estimated TB incidence rates of 483 (95% CI: 370–611) and 425 (95% CI: 323–540) per 100 000 population, respectively (**Fig. 2**). Fiji had the highest estimated number of TB cases (*n* = 590), followed by Kiribati (*n* = 510), the Solomon Islands  (*n* = 450) and the Marshall Islands (*n* = 290). These four PICs accounted for 78% of the total cases in the subregion. American Samoa, Samoa and Wallis and Futuna had an estimated TB incidence rate of < 10 cases per 100 000 population in 2020.

[**insert** Figure 2]

### TB cases

**Fig. 3:**
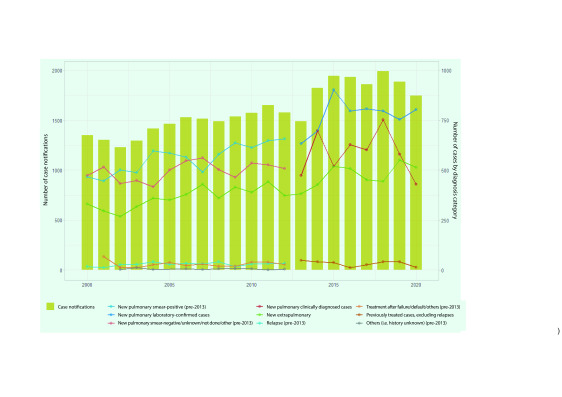
Number of TB notifications by diagnosis category in Pacific island countries and areas, 2000–2020

The number of reported TB cases (new and relapse) in the subregion has increased over the last two decades, ranging from 1229 in 2002 to 1991 in 2018 (**Fig. 3**). Between 2000 and 2020, the number of TB cases increased by 29% to 1746 in 2020. The number of bacteriologically confirmed or smear-positive TB cases increased by 66%, from 485 in 2000 to 804 in 2020. The number of clinically diagnosed or smear-negative cases ranged from 474 in 2000 to 429 in 2020, with some fluctuations. The number of EPTB cases increased by 55%, from 331 in 2000 to 513 in 2020. Among new and relapse PTB cases, 65% (*n* = 804/1233) were bacteriologically confirmed in 2020, with clinically diagnosed cases accounting for the rest. The proportion of EPTB among all TB cases was 29% (*n* = 513/1746) in 2020.

[**insert** Figure 3]

**Fig. 4:**
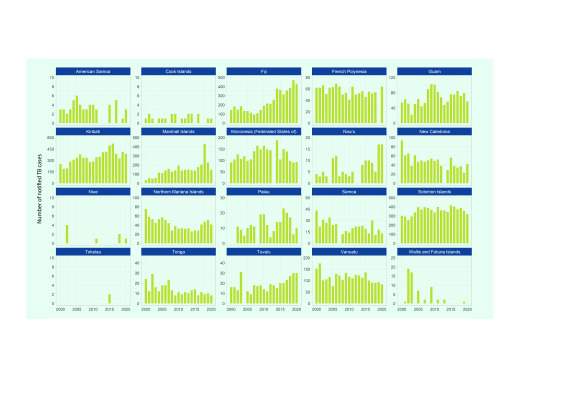
Number of TB notifications (new and relapse) by year in Pacific island countries and areas that provided data, 2000–2020

By country and area, between 2000 and 2020, TB cases increased in Fiji (from 144 to 431), Kiribati (from 252 to 385) and the Marshall Islands (from 34 to 147) (**Fig. 4**). TB cases decreased between 2000 and 2020 in New Caledonia, the Northern Mariana Islands, Samoa, Tonga, Vanuatu and Wallis and Futuna, with some fluctuations observed over the period. In Samoa, TB cases decreased by 70% (from 43 to 13) over the same period.

[**insert** Figure 4]

### Age and sex distribution

**Fig. 5:**
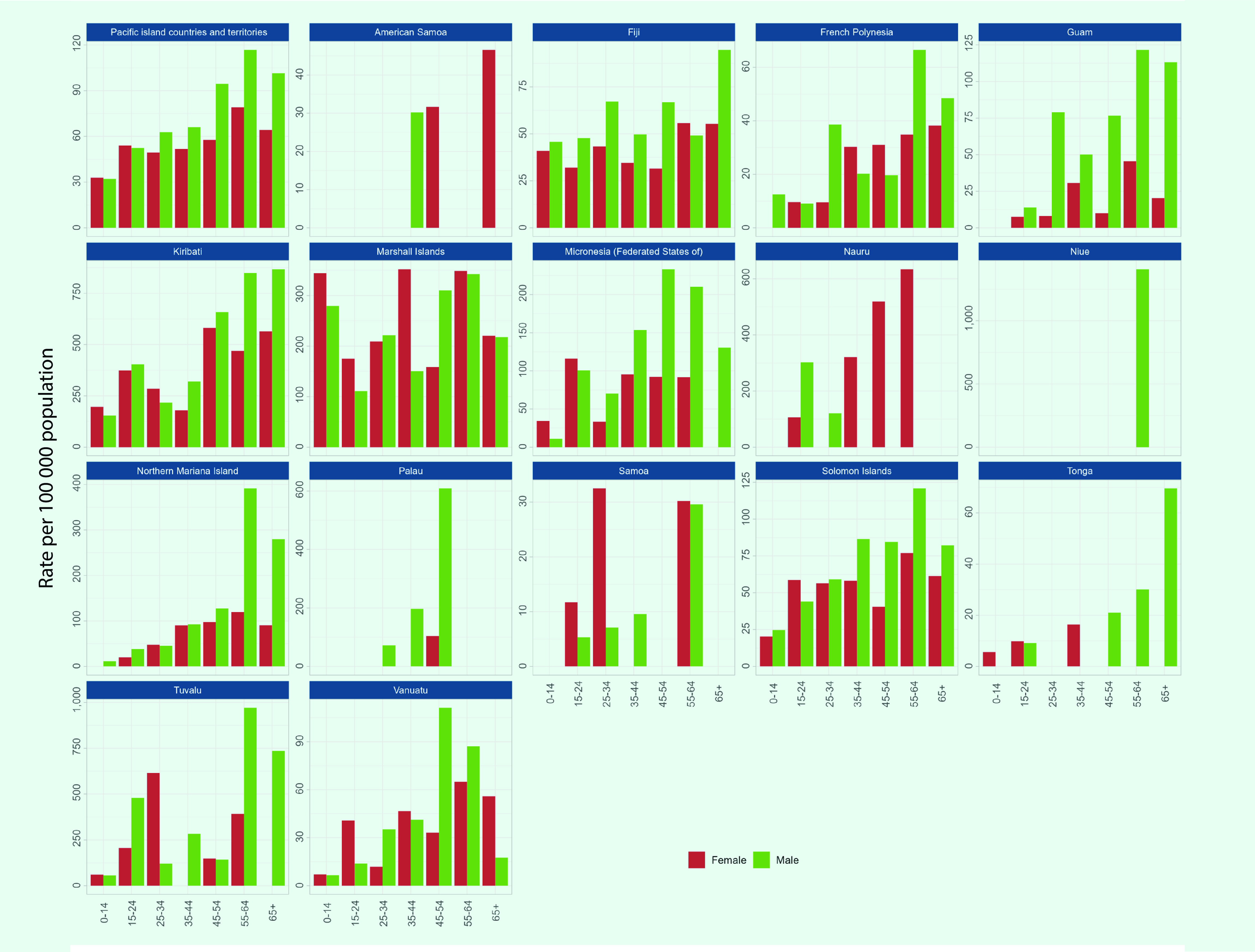
Age and sex distribution of TB notifications (new and relapse) per 100 000 population in the subregion overall and in Pacific island countries and areas that provided data, 2020

TB cases with data on age and sex were reported from 16 PICs in 2020. Among these 1695 cases, 56%  (*n* = 945) were male, 19% (*n* = 328) were children aged < 15 years and 8% (*n* = 141) were older adults aged ≥ 65 years. In the subregion, the notification rate was higher for older age groups, with the highest rate for both males and females aged 55–65 years (117 cases and 79 cases per 100 000 population, respectively)  (**Fig. 5**). Notification rates were higher in males aged ≥ 25 years, while they were higher for females among children and younger adults.

High TB notification rates were reported for children aged < 15 years for both sexes in the Marshall Islands, at over 250 per 100 000 population. Also, the Marshall Islands had the highest proportion of TB cases in children at 37% (*n* = 54/147). In Kiribati and Tuvalu, rates of over 800 per 100 000 population were reported for males aged 55–64 years (**Fig. 5**).

[**insert** Figure 5]

### Diagnosis category and type of TB

**Fig. 6:**
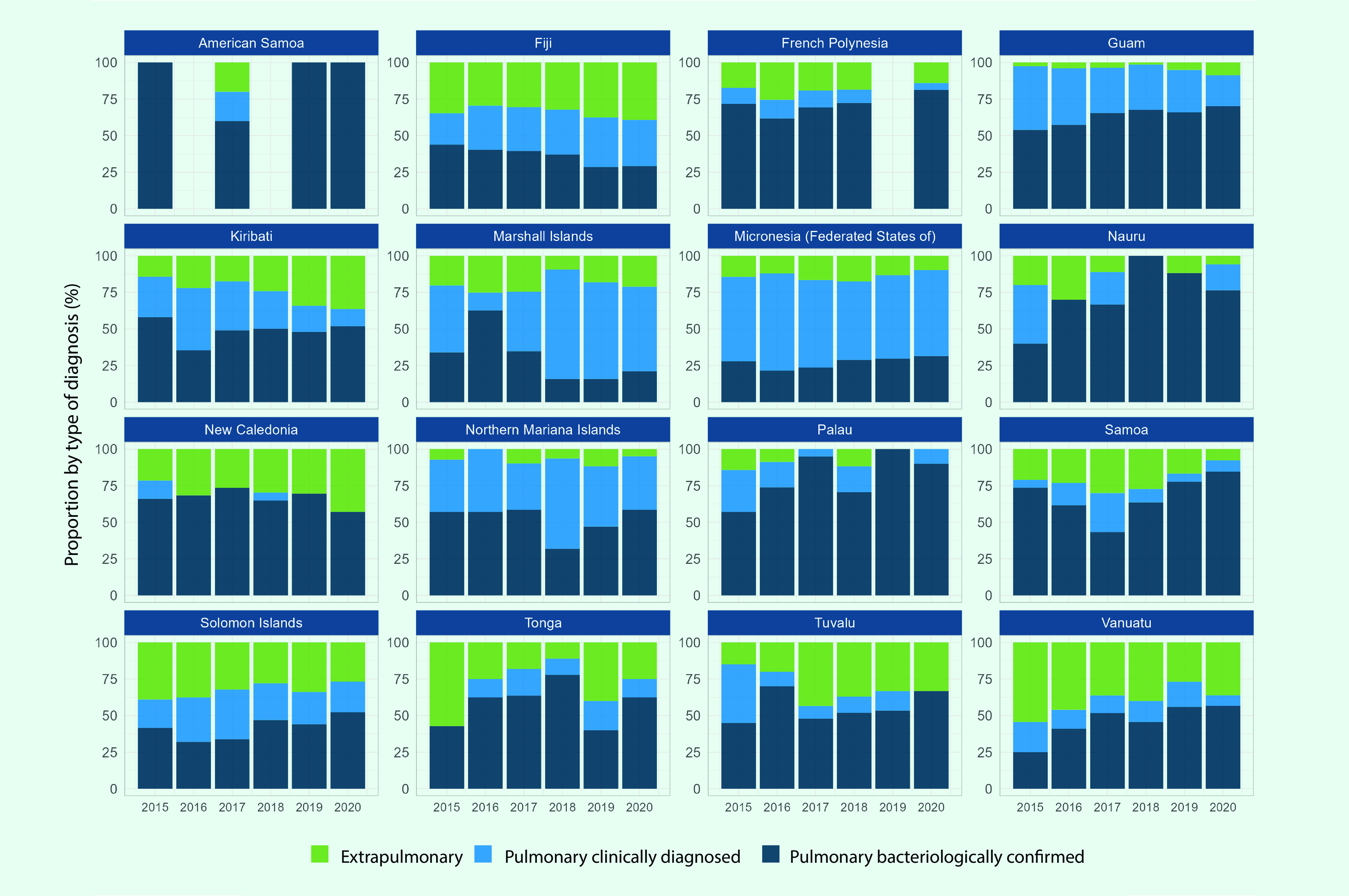
Number of TB notifications by year and type of diagnosis in Pacific island countries and areas that provided data, 2015–2020

Diagnosis category and type of TB were reported from  20 PICs, four of which were excluded from the analysis as they reported less than five cases cumulatively between 2015 and 2020. Bacteriological diagnosis was more common compared to clinical diagnosis in 2020 in 13 of the 16 PICs that reported diagnosis category (**Fig. 6**) with 58% of PTB cases in 2020 being bacteriologically confirmed (*n* = 4809/8302). There were higher proportions of clinical diagnosis for PTB cases in the Federated States of Micronesia (69% from 2015 to 2020), Fiji (53% from 2019 to 2020) and the Marshall Islands (80% from 2018 to 2020). In the Marshall Islands, the number of PTB cases that were clinically diagnosed increased from 83 in 2017 to 321 in 2018. The proportion of PTB among new and relapse cases in the subregion between 2015 and 2020 was 78% (8752/11 189).

[**insert** Figure 6]

### Drug-resistant TB

**Fig. 7:**
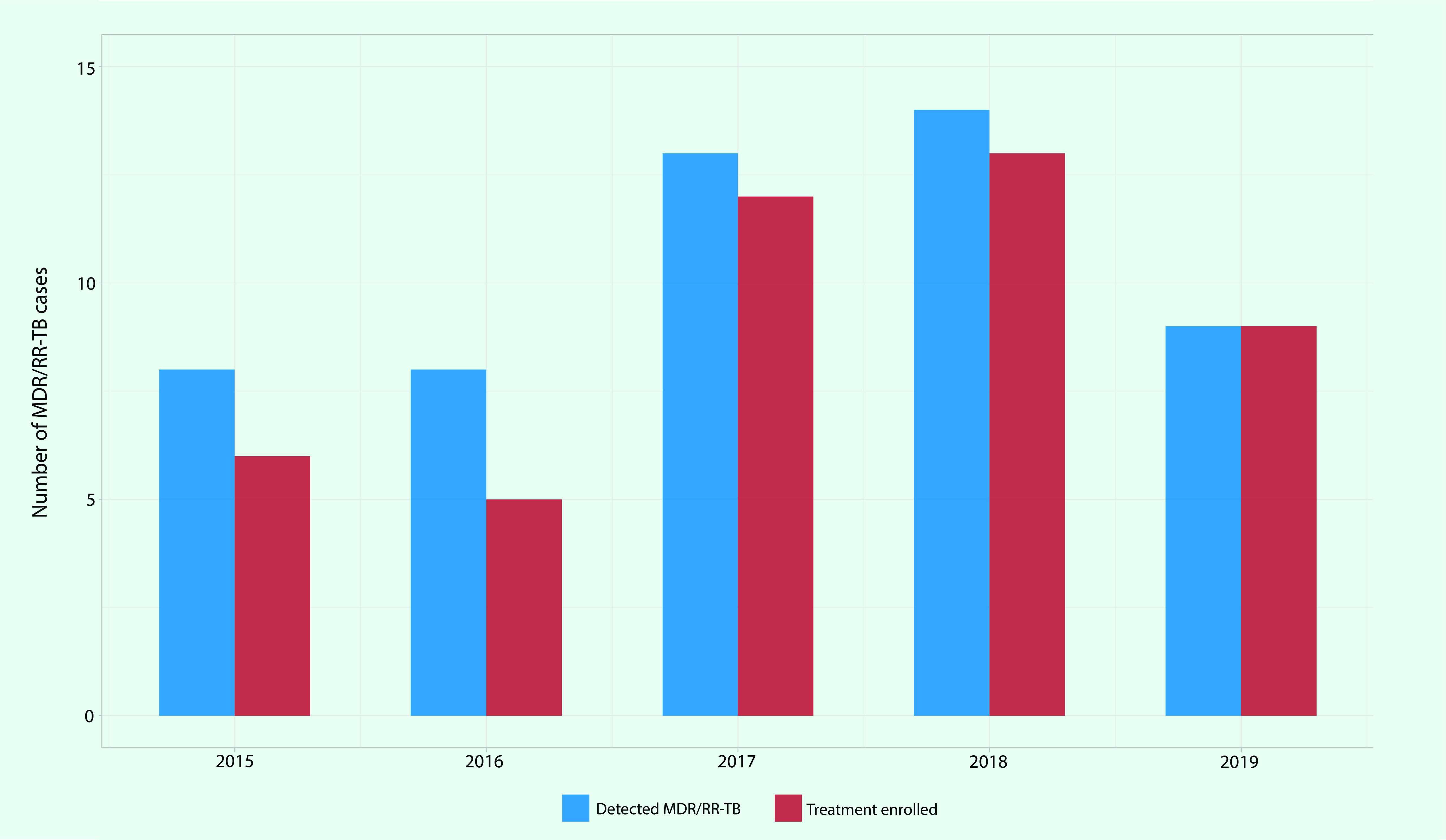
Number of MDR/RR-TB cases reported and enrolled in MDR-TB treatment in Pacific island countries and areas that provided data, 2015–2019

MDR/RR-TB cases were reported from 15 PICs between 2015 and 2019, while five PICs (the Cook Islands, Nauru, Niue, Tonga and Wallis and Futuna) reported no cases. There were 52 MDR/RR-TB cases detected, of which 87% (*n* = 45) were enrolled in MDR-TB treatment. The number of MDR/RR-TB cases fluctuated between eight and 14 per year with nine cases in 2019 (**Fig. 7**). The highest number of MDR/RR-TB cases was reported in Kiribati with nine detected and treated.

[**insert** Figure 7]

### Indicators of collaborative TB/HIV activities

Data on known HIV status and HIV prevalence were available from 18 PICs, and data on cases of TB and HIV coinfection receiving antiretroviral therapy (ART) were available from eight PICs. The prevalence of HIV among TB cases and the proportion of TB and HIV coinfection receiving ART have been sparsely recorded or reported by the majority of PICs. Data on PLHIV eligible for TB preventive treatment and PLHIV who were started on the treatment were not available in any of the PICs.

**Fig. 8:**
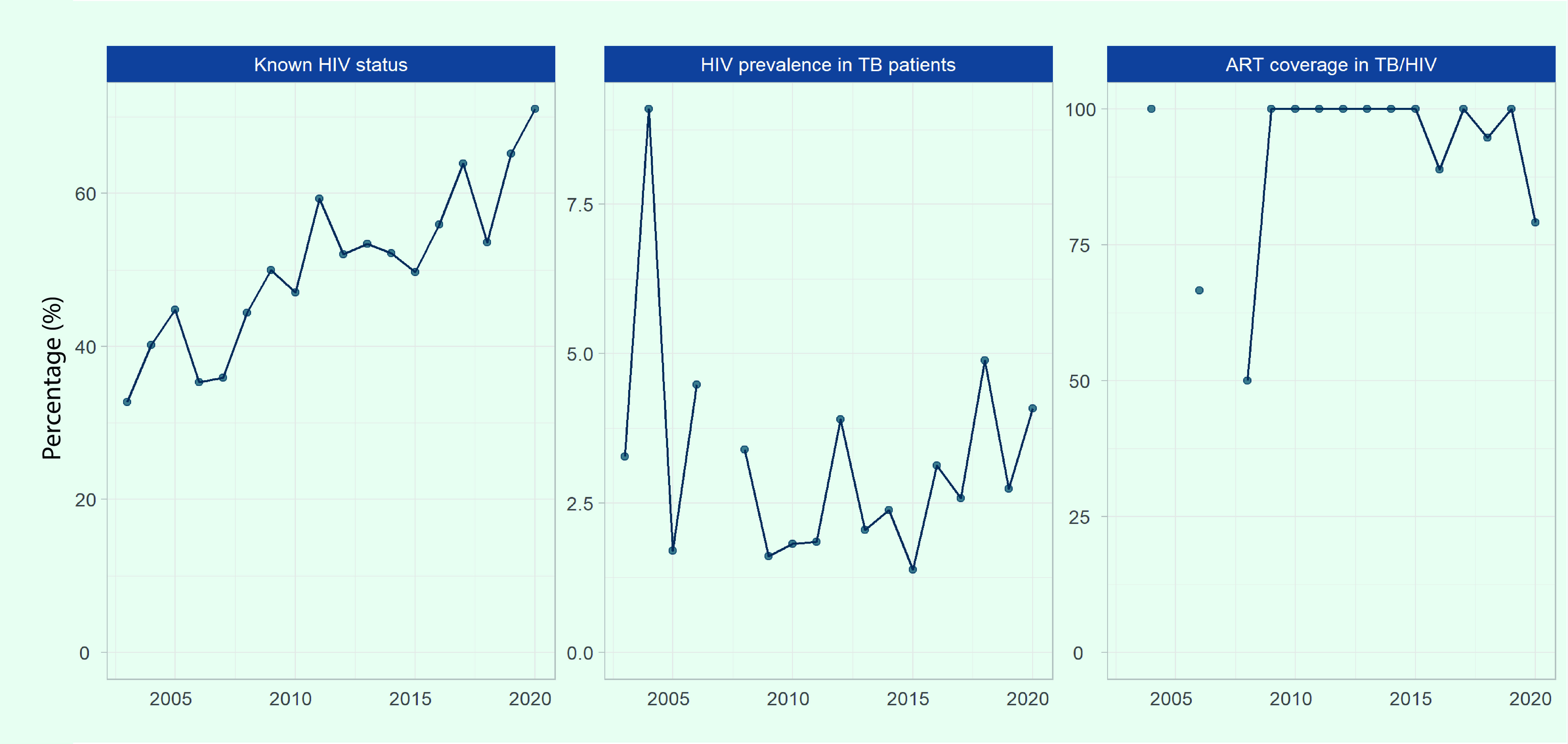
Known HIV status, HIV prevalence in TB patients and antiretroviral therapy coverage for TB/HIV patients in Pacific island countries and areas that provided data, 2003–2020

The proportion of TB cases with HIV status recorded in the subregion increased from 33% in 2003 to 71% in 2020 (Fig. 8). This proportion varies by PIC, with 100% reported in Fiji, the Northern Mariana Islands, Palau and Samoa in 2020, and less than 50% reported in French Polynesia (43%), Kiribati (42%) and the Solomon Islands (38%).

The number of reported TB cases coinfected with HIV was low in the subregion, with 94 cases reported between 2015 and 2020 (16 cases per year on average, 0.8% [*n* = 94/11 311] of total notified cases). The prevalence of HIV among TB cases who were tested for HIV has remained below 5%, except for 2004 when 9% (*n* = 2/22) was observed due to a small number of cases tested and detected. Despite the decrease in HIV prevalence among TB cases in the subregion, there has been an increase in Fiji, from a low of 0.8% in 2005 to 6.2% in 2019 and 5.0% in 2020.

The proportion of TB and HIV coinfected cases receiving ART was 79% in 2020, a decrease from over 88% reported between 2009 and 2019 (**Fig. 8**). Fiji submitted 81% (*n* = 109/135) of the data on ART.

[**insert** Figure 8]

### Treatment outcomes

**Fig. 9:**
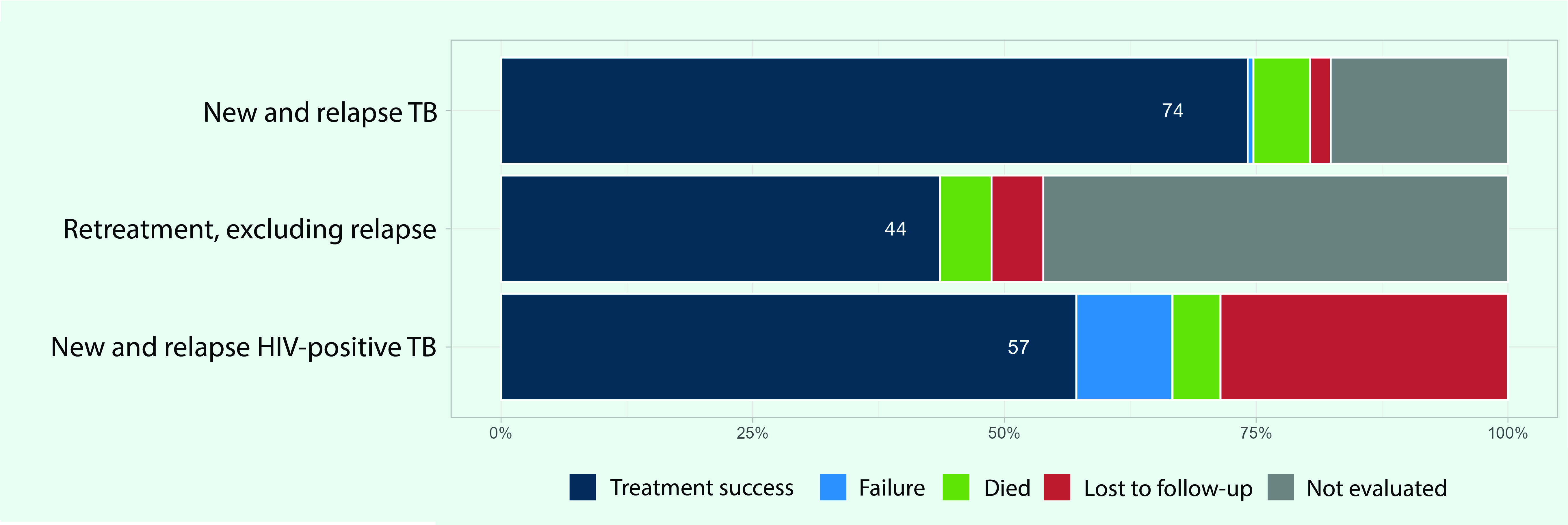
TB treatment outcomes by patient category in Pacific island countries and areas that provided data, 2019

Treatment outcomes were reported from 19 PICs for the 2019 patient cohort, of which three PICs reported no cases. The treatment success rate was 74% for new and relapse cases, 44% for retreatment cases (excluding relapse) and 57% for HIV-positive TB cases (**Fig. 9**). Approximately 18% of the new and relapse cases were not evaluated on their treatment outcomes.

[**insert** Figure 9]

**Fig. 10:**
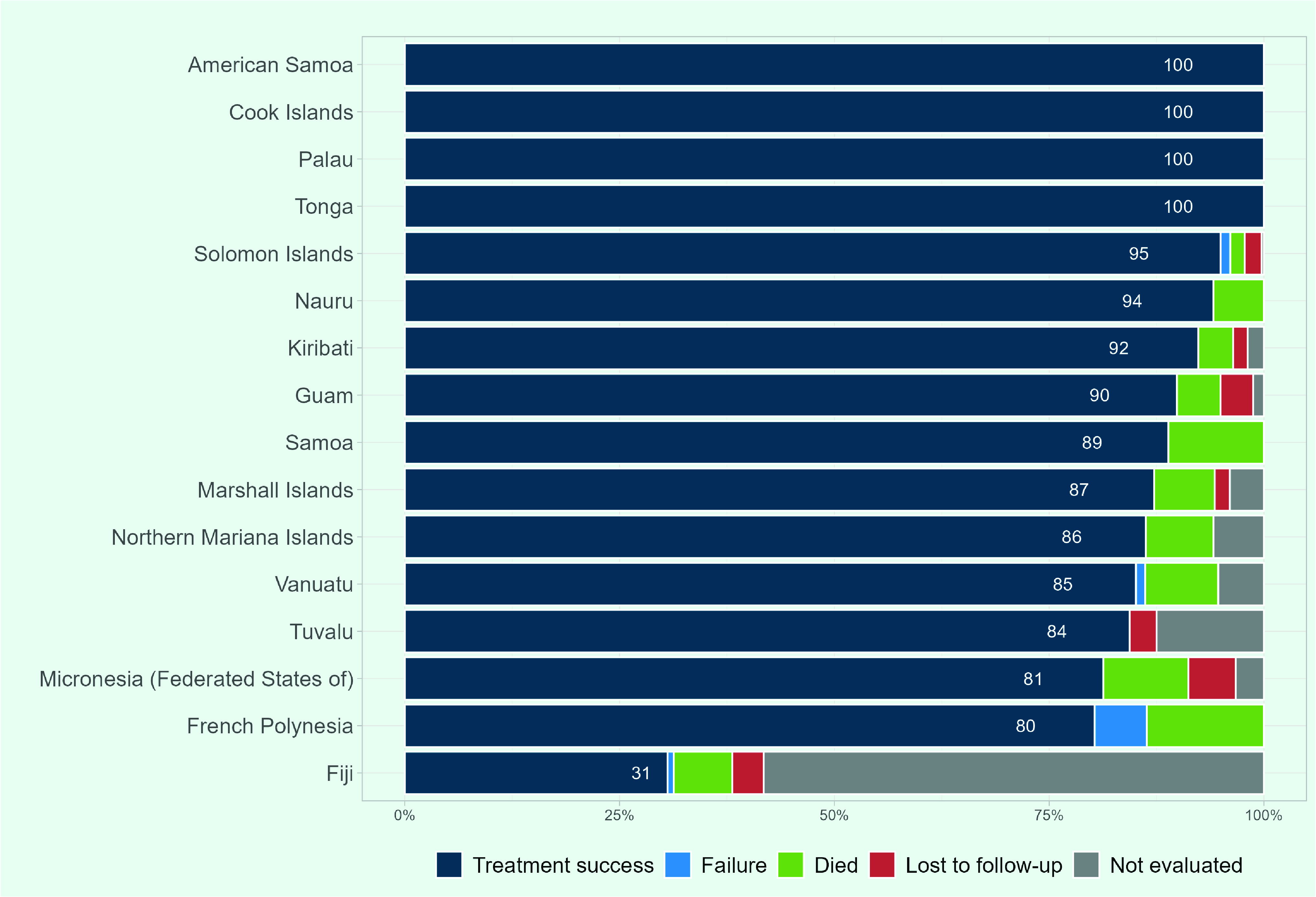
Treatment outcomes among new and relapse TB cases in Pacific island countries and areas that provided data, 2019

Treatment success rates of 90% or more were reported in eight PICs for the 2019 patient cohort, and 100% in American Samoa, the Cook Islands, Palau and Tonga (**Fig. 10**). Eight of the 16 PICs had treatment success rates of less than 90%, while four of the PICs reported less than 85%: Tuvalu, the Federated States of Micronesia, French Polynesia and Fiji reported 84%, 81%, 80% and 31%, respectively. In Fiji, 58% of cases (*n* = 333/572) were either not evaluated or did not have their treatment outcome recorded.

[**insert** Figure 10]

### Risk factors

Data on the estimated proportion of TB cases attributable to alcohol abuse, diabetes mellitus, smoking and undernutrition are available for 12, 13, 10 and seven PICs, respectively. These proportions were 17%  (*n* = 226/1299) for smoking, 16% (*n* = 206/1297) for undernutrition, 11% (*n* = 154/1392) for alcohol abuse and 9% (*n* = 133/1509) for diabetes mellitus. The proportion of cases attributable to diabetes mellitus was higher in PICs compared to the proportion in the entire Western Pacific Region at 6%. Conversely, the proportion of cases attributable to the other risk factors was almost the same or lower in PICs than in the Region overall, where it was 17% for smoking, 20% for undernutrition and 16% for alcohol abuse.

Among the PICs, the highest proportions of TB cases attributable to smoking and undernutrition were estimated in the Solomon Islands at 19% (*n* = 61/170) and 31% (*n* = 100/321), respectively. Of the 13 PICs with data available for diabetes mellitus, the proportion was highest in Nauru at 12% (*n* = 2/17) and in the Marshall Islands at 12% (*n* = 17/147), followed by Kiribati at 10% (*n* = 39/385). Of the 12 PICs with data available for alcohol abuse, Vanuatu reported the highest proportion at 13% (*n* = 11/83), followed by the Solomon Islands at 12% (*n* = 38/321).

### TB preventive treatment

In 2020, of the 19 PICs that reported case notification data, 53% reported the number of household contacts of new and relapse PTB cases that were bacteriologically confirmed and started on TB preventive treatment. In these PICs, the number of contacts identified totalled 3049 in 2020. Of those, 38% (*n* = 1159/3049) started TB preventive treatment, 19% (*n* = 220/1159) of whom were children aged under 5 years.

## Discussion

This analysis showed increases in the estimated TB incidence rates and the number of TB cases and deaths in the Pacific islands subregion between 2000 and 2020. There was an increased proportion of bacteriological confirmation for TB diagnosis, increased HIV testing coverage in TB patients and sustained high ART coverage in the small number of patients requiring ART. The results also highlighted a high proportion of TB cases in the younger population, poor treatment outcomes in some PICs, and a large number of TB cases with underlying diabetes mellitus and other risk factors. PICs have diverse TB burdens, with Fiji, Kiribati, the Marshall Islands and the Solomon Islands all considered high TB burden countries in the Pacific, which was confirmed by the results of this analysis. In these PICs, increased efforts to strengthen national TB programmes and secure domestic and external support are essential.

The number of TB notifications increased in Fiji, Kiribati and the Marshall Islands over the last two decades. Increases in notifications can be driven by various factors including the improvement of TB screening, implementation of active case finding activities, (*14*) improved recording and reporting, (*15*) and increased TB transmission within the community. In Fiji, for example, the TB notification rate in the younger population increased in the early 2010s, which might indicate increased community transmission. (*16*) At 19%, the proportion of TB cases in children from the subregion in 2020 was higher than the WHO Western Pacific Region at 4% and the global proportion at 12%. This underscores the importance of intensifying household contact investigation to cut the chain of transmission. In the Marshall Islands, case notifications sharply increased in 2018, mostly in clinically diagnosed cases. This is probably due to population-based screening programmes for latent and active TB which were conducted on Ebeye and Majuro islands in 2017 and 2018, covering nearly 75% of the national population. (*17*) Such population-based mass screening and treatment of latent and active TB has the potential to reduce the incidence of TB in a short period (*17*) and could be a key intervention in advancing efforts to eliminate TB in PICs, given their geographical isolation and limited population size. (*18*)

The number of reported MDR/RR-TB cases has increased but fluctuated over the last two decades, as observed in the Western Pacific Region where small numbers and irregular MDR-TB caseloads were reported in the selected PICs. (*19*) Since this report in 2014, Kiribati has detected its first MDR/RR-TB cases with three each year since 2017. (*7*) Other areas, such as French Polynesia and Guam, have consistently reported MDR/RR-TB cases since 2015. These diagnoses may reflect improved surveillance systems and the expanded use of Xpert MTB/RIF. The number of Xpert testing sites has increased over time in Fiji, Kiribati, the Solomon Islands and Vanuatu, (*11*) which may have contributed to the early detection of drug-resistant TB cases and improved the proportion of bacteriological confirmation in TB diagnoses. Treatment outcomes among MDR/RR-TB cases were not analysed in this report as the data were not available. This might be due to a lack of follow-up, recording or reporting.

HIV prevalence among TB cases in the subregion was low, although an increased prevalence was reported in Fiji. This is probably due to an increase in new HIV infections among the general population in Fiji, which increased from 0.15 per 1000 population among adults aged 15–49 in 2010 to 0.32 per 1000 population in 2021. (*20*) It is strongly recommended that PLHIV be systematically screened for TB disease at each visit to a health facility. (*21*) Proper management of HIV, including initiation and continuation of ART, is essential to prevent the development of TB disease among PLHIV. (*22*) Simultaneously, continuous HIV screening among TB patients and timely initiation of treatment among HIV coinfected cases is imperative. (*23*)

Management of diabetes mellitus should be highlighted in the subregion, considering its high prevalence among the general population. Of the 16 PICs with these data available, nine reported a prevalence higher than 20% in 2021. (*24*) Among them, six were ranked in the top 10 countries with the highest prevalence of diabetes mellitus, with French Polynesia exceeding 25%. For optimal management of diabetes mellitus, it is important to ensure accessibility to primary health-care services and community-level health promotion programmes targeting younger age groups. (*25*) Additionally, close collaboration and coordination between multiple disease programmes and sectors are crucial in addressing TB and its risk factors simultaneously. (*8*)

Treatment success for new and relapse TB cases in the PICs that reported the data was 74%. This is low when compared to the whole Western Pacific Region where it has been approximately 90% over the past decade, (*5*) and to the global data where it was 86% in 2019. (*1*) The treatment success rate was low in Fiji due to the high proportion of unevaluated cases, which may be attributable to the recent transition from the Global Fund-supported vertical programme, resulting in limited human resources responsible for recording and reporting. There are several more PICs with success rates below 90%. These rates might be attributable to delays in diagnosis and treatment due to limited access to health facilities that provide TB services, coupled with the unique geographical challenges of PICs. This may be underpinned by fear of financial burden and stigma. For example, in Vanuatu, the majority of TB cases report having experienced stigma after diagnosis, and more than half of patients first consult a traditional healer due to the cost and distance to health facilities. (*26*)

This analysis has several limitations. There is great uncertainty in the estimated incidence rates and number of deaths as evidenced by the wide range of confidence intervals. First, estimated TB incidence is calculated based on case notifications with a standard adjustment or adjustments made based on expert opinions, which might not reflect the actual incidence. (*12*) Most PICs have their mortality estimates indirectly derived from the incidence. In settings with a limited number of notified cases, modelled estimates are less appropriate compared to other methods of estimation that are based on surveys due to the strong stochasticity of the reported cases. Second, the observed increase in the estimated number of deaths in 2020 might not reflect the actual number of deaths in the subregion. While the modelled estimate accounted for the shortfall in TB case detection due to the disruption of health services caused by the coronavirus disease (COVID-19) pandemic, most PICs reported no or limited COVID-19 community transmission in 2020. Therefore, extensive TB service disruption was unlikely. Data submitted by the PICs are often incomplete. Notably, data on age and sex distribution and HIV test results were missing for 71% and 76% of the cohort, respectively. Hence, some data shown in this report might not represent the subregion.

In conclusion, the number of notified TB cases in PICs has increased over the years, with signs of ongoing active community transmission, and the burden is distributed unevenly across countries and areas. The ongoing effort to scale up laboratory services is an achievement, and the implementation of community-based screening appears promising especially in small island settings. Greater effort and investment are needed to reach the unreached population including those who have risk factors and socioeconomic and geographical disadvantages. Furthermore, strengthening routine contact investigation, scaling up TB preventive treatment, and ensuring proper management of TB cases and comorbidities through a patient-centred approach are priority interventions to end TB in the context of PICs.
